# Study protocol: Mother and Infant Nutritional Assessment (MINA) cohort study in Qatar and Lebanon

**DOI:** 10.1186/s12884-016-0864-5

**Published:** 2016-05-04

**Authors:** Farah Naja, Lara Nasreddine, Al Anoud Al Thani, Khaled Yunis, Michael Clinton, Anwar Nassar, Sara Farhat Jarrar, Patricia Moghames, Ghina Ghazeeri, Sajjad Rahman, Walaa Al-Chetachi, Eman Sadoun, Nibal Lubbad, Zelaikha Bashwar, Hiba Bawadi, Nahla Hwalla

**Affiliations:** Department of Nutrition and Food Sciences, American University of Beirut, Beirut, Lebanon; Health Promotion and Non Communicable Disease Prevention Division, Supreme Council of Health, Al Rumaila West, Doha, Qatar; Department of Pediatrics and Adolescent Medicine, American University of Beirut Medical Center, Beirut, Lebanon; Social and Behavioral Institutional Review Board, American University of Beirut, Beirut, Lebanon; Department of Obstetrics and Gynecology, American University of Beirut Medical Center, Beirut, Lebanon; Department of Pediatrics, Al Ahli Hospital, Doha, Qatar; Department of Healthcare Quality Management, Supreme Council of Health, Doha, Qatar; Department of Family Medicine, Primary Health Care Corporation, Doha, Qatar; Department of Health Sciences, Qatar University, Doha, Qatar; Faculty of Agricultural and Food Sciences, American University of Beirut, Beirut, Lebanon

**Keywords:** Nutrition, Pregnancy, Growth, Development, Non-communicable diseases, Cohort, Middle East and North Africa, Feeding practices

## Abstract

**Background:**

The Middle East and North Africa region harbors significant proportions of stunting and wasting coupled with surging rates of non-communicable diseases (NCDs). Recent evidence identified nutrition during the first 1000 days of life as a common denominator not only for optimal growth but also for curbing the risk of NCDs later in life. The main objective of this manuscript is to describe the protocol of the first cohort in the region to investigate the association of nutrition imbalances early in life with birth outcomes, growth patterns, as well as early determinants of non-communicable diseases. More specifically the cohort aims to1) examine the effects of maternal and early child nutrition and lifestyle characteristics on birth outcomes and growth patterns and 2) develop evidence-based nutrition and lifestyle guidelines for pregnant women and young children.

**Methods/design:**

A multidisciplinary team of researchers was established from governmental and private academic and health sectors in Lebanon and Qatar to launch the Mother and Infant Nutritional Assessment 3-year cohort study. Pregnant women (*n* = 250 from Beirut, *n* = 250 from Doha) in their first trimester are recruited from healthcare centers in Beirut, Lebanon and Doha, Qatar. Participants are interviewed three times during pregnancy (once every trimester) and seven times at and after delivery (when the child is 4, 6, 9, 12, 18, and 24 months old). Delivery and birth data is obtained from hospital records. Data collection includes maternal socio-demographic and lifestyle characteristics, dietary intake, anthropometric measurements, and household food security data. For biochemical assessment of various indicators of nutritional status, a blood sample is obtained from women during their first trimester. Breastfeeding and complementary feeding practices, dietary intake, as well as anthropometric measurements of children are also examined. The Delphi technique will be used for the development of the nutrition and lifestyle guidelines.

**Discussion:**

The Mother and Infant Nutritional Assessment study protocol provides a model for collaborations between countries of different socio-economic levels within the same region to improve research efficiency in the field of early nutrition thus potentially leading to healthier pregnancies, mothers, infants, and children.

**Electronic supplementary material:**

The online version of this article (doi:10.1186/s12884-016-0864-5) contains supplementary material, which is available to authorized users.

## Background

Nutrition has important implications on people’s health throughout their life cycle particularly during periods of rapid growth and development, namely during pregnancy and early childhood. This period is referred to as “the first 1000 days of life” [1], during which maternal and child nutrition, including both under- and over-nutrition, has been found to affect optimal growth and disease susceptibility later in life.

The association between maternal and child malnutrition with growth and development has been well documented in the literature. A review of evidence from 35 studies revealed a direct association between limited maternal weight gain during pregnancy and both impaired fetal growth and low birth weight [2]. Also, excess maternal weight gain was found to be correlated with high birth weight and fetal growth (large-for-gestational age) [2–4] as well as adverse cardiometabolic profile in offspring [5]. In addition to maternal weight gain, maternal diet composition and micronutrient status during pregnancy seem to be associated with birth outcomes and child health status. During pregnancy, a low-protein diet has been associated with low birth weight [6, 7], while a diet rich in fat was shown to affect adipocyte metabolism, fetal growth, and fat mass in offspring [8–11]. Maternal anemia and deficiency of iron, folate, or vitamin B12 during pregnancy have also been associated with an increased risk of fetal growth restriction, premature birth, and low birth weight [12–17].

The first 1000 days of life encompass, besides the period of gestation, early childhood up to two years of age, during which feeding practices (including breastfeeding and complementary feeding) were found to have a major influence on optimal growth and mortality [18–22].

In addition to its critical role in growth and development, nutrition during the first 1000 days of life has recently been shown to play a pivotal role in the etiology of non-communicable diseases (NCDs) later in life. The concept of adult disease originating early in life was first described by Forsdahl in 1977 [23], and was further developed by Barker and colleagues [24]. This concept, often coined as ‘fetal or metabolic programming’, is explained as the process occurring during critical periods of development when adaptive alterations to the structure and/or function of various systems and key body organs occur, in response to environmental stressors, such as nutritional disturbances [25]. Consequently, the first 1000 days of a child’s life is considered as a critical ‘window of opportunity’ for intervention and prevention of later-onset NCDs [18, 26, 27]. The recent scientific literature has witnessed a plethora of studies investigating the effects of nutrition during this period of life on early determinants of NCDs. A few meta-analyses and review reports have shown that breastfeeding (as compared to bottle-feeding) [28–32], as well as the duration of breastfeeding [33, 34] are protective against obesity and NCDs later in life, through inducing slower and more linear patterns of growth during infancy [21, 33, 35, 36]. Additionally, the early introduction of complementary food has been found to be associated with childhood obesity as well as adulthood disease risk [37, 38].

Most of the evidence for the critical role of nutrition during the first 1000 days of life was drawn from studies conducted in developed countries. Recently, two Lancet series published in 2008 and 2013 were dedicated to examine maternal and child nutrition in low- and middle- income countries, and have described the results of cohort studies conducted in Brazil, Guatemala, India, the Philippines, and South Africa. Up to this date, no such cohort studies examining mother and child nutrition and their effect on health and disease have taken place in the Middle East and North African (MENA) region.

In several countries of the MENA region, there is a coexistence of stunting and wasting, with surging rates of overweight, obesity, and NCDs. On one hand, it is estimated that 15 % of the global burden of mortality among newborns and young children occurs in countries of the Eastern Mediterranean region [39], with malnutrition accounting for an estimated 50 % of deaths among the region’s underfive population [40]. On the other hand, rates of overweight and obesity in the under-five population in certain countries of the MENA region are comparable and even higher than rates in developed countries [41, 42]. Moreover, an alarmingly high prevalence of NCDs also exists in the Eastern Mediterranean region [43, 44]. According to World Health Organization (WHO) estimates, the percentage of deaths inflicted by NCDs is estimated at 60 % in the MENA region [39]. Given the importance of nutrition in influencing early growth and development, as well as later-susceptibility to chronic disease, there exists an urgent need for evidence-based and culture-specific nutrition interventions targeting both young children and women of child-bearing age, especially during pregnancy [45]. To move this agenda forward, collaboration was initiated between Qatar and Lebanon to launch the first mother and child cohort study, examining the effect of maternal and young child nutrition and lifestyle characteristics on birth outcomes and growth patterns. Qatar and Lebanon are two Arab countries of the MENA region that can generally represent fossil fuel-exporter and fossil fuel-importer countries, respectively. Qatar is an economically fast-growing country of the Gulf Cooperation Council (GCC), undergoing a nutrition transition following continued growth in population, per-capita income, and wealth [46, 47]. Lebanon, on the other hand, is a middle-income country also undergoing nutritional and demographic transitions, following an increasing urbanization rate, life expectancy, and westernization of lifestyle [48]. In Qatar, significant proportions of stunting (11.6 %), underweight (4.8 %) and wasting (2.1 %) [49] are coupled to a 69 % rate of deaths caused by NCDs [50]. Furthermore, Qatar has recorded the second highest obesity prevalence rates in the region after Kuwait, with around 40 % of the adult Qatari population being obese [51]. Not only are obesity figures high among Qatari adults, they have reached disturbing levels among children (21.5 %) and adolescents as well (4.9 %) [52]. In Lebanon, similar to Qatar, proportions of stunting (16.5 %), underweight (4.2 %), and wasting (6.6 %) [49] co-exist with surging rates of NCDs, with the percentage of deaths attributed to these diseases reaching as high as 85 % [50]. The prevalence of obesity in the Lebanese population has increased by almost 2 folds in the past decade, with findings from a recent nationally representative study revealing obesity rates of 28.2 % among adults and 10.9 % among children and adolescents [53].

### Objectives

The main objective of the manuscript is to describe the protocol of the mother and child cohort study examining the effects of maternal and young child nutrition and lifestyle characteristics on birth outcomes and growth patterns in Lebanon and Qatar; the first cohort study in the MENA region to investigate the association of nutrition imbalances early in life with birth outcomes, growth patterns, as well as early determinants of NCDs. The goal of this cohort study is to promote balanced nutrition and health during the first 1000 days of life through developing evidence-based country-specific nutrition and lifestyle guidelines for pregnant women and young children in Lebanon and Qatar. These guidelines will constitute the foundation of effective interventions to ensure optimal growth and development of children as well as to curb the risk of NCDs later in life. Specific objectives of this cohort study include:

- Prospective evaluation of the influence of nutritional status and lifestyle factors during pregnancy on birth outcomes and growth patterns of young children
- Identification of faulty child feeding practices and evaluation of their effect on growth patterns of young children
- Determination of the effect of household food insecurity on maternal nutrition, birth outcomes, and nutritional status of young children

## Methods/design

### Ethical approval

The MINA cohort protocol was approved by the Institutional Review Board (IRB) at the American University of Beirut (Protocol ID: NUT. FN. 12) and at the Primary Health Care Corporation in Qatar (Protocol ID: PHCC/RC/15/04/006). Collaboration between investigators in two different countries brought a higher level of complexity to the ethical oversight of human subject research. The challenge was to ensure sufficient ethical oversight of the study, while respecting the interests, status, and procedures of each of the ethical review bodies that have jurisdiction over the study conduct. Participants are asked to provide signed consent to participate in the study at the time of recruitment. In addition to the main consent form, participants are given the option to give a written consent for obtaining delivery and birth data from medical records, sharing the collected data with researchers that are not part of the study, as well as storage and use of left-over samples for future research. Subjects are informed that they can still participate in the study even if they decline consenting for the additional options. Participants are given a copy of the signed consent form.

### Study design

This study is a longitudinal three-year cohort study of pregnant women and their children residing in Lebanon and Qatar. The study population includes all pregnant women attending the obstetrics and gynecology (OBGYN) clinics of the following health-care centers in Lebanon and in Qatar:

- The American University of Beirut Medical Center (AUBMC) and primary health centers in Beirut. These health centers represent the both private and governmental hospitals in Beirut, Lebanon, respectively.
- Primary Health Care Corporation (PHCC) clinics in Doha, Qatar. PHCC currently operates 21 primary health care centers in Qatar, 13 of which are located in Doha city.

### Study participants

Study participants are selected according to the below set inclusion and exclusion criteria.

Inclusion criteria for pregnant women:

- Within the first trimester of pregnancy (between 0-13 weeks of gestation)
- Pregnant with a singleton
- Of Lebanese or Qatari nationality and non-Qatari nationality living in Qatar for more than 5 years
- Not planning on permanently leaving either of the countries during the timeframe of the study
- Absence of a chronic illness preconception (diabetes, hypertension, kidney disease, cancer, and other chronic diseases or infections such as autoimmune disorders, human immunodeficiency virus, and hepatitis)

Exclusion criteria: Pregnant women are excluded from the study if they were:

- Carrying twins or multiple babies
- Had a history of a chronic illness
- Had a history of multiple gestations (twins or triplets)
- Had previously given birth to babies with physical malformations, mental retardations, and/or inborn errors of metabolism

### Study protocol

**Fig. 1:**
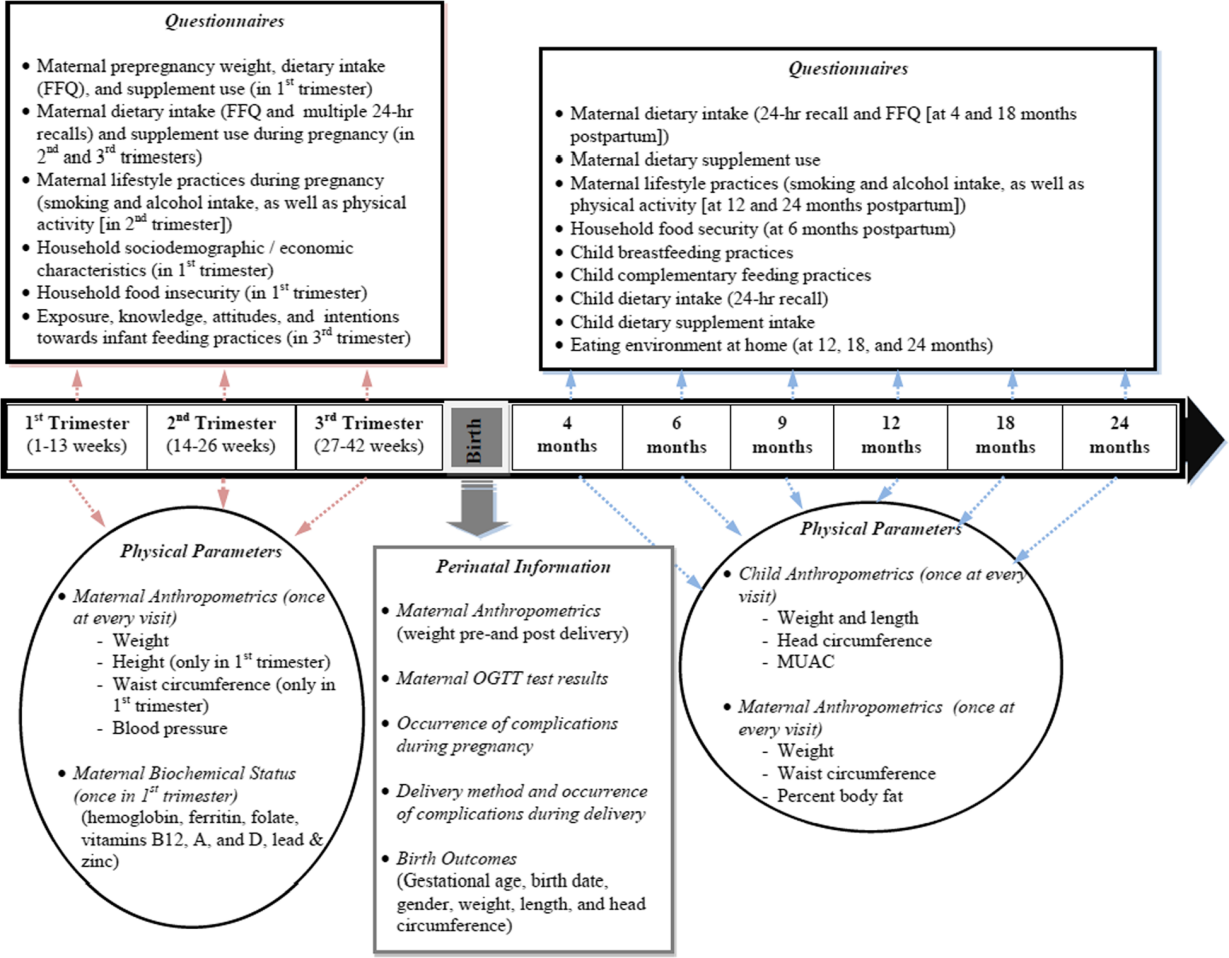
Assessment timeline of the MINA cohort study

The study protocol is summarized in an assessment timeline, as outlined in Fig. 1, whereby a total of 9 visits take place (3 visits during each trimester of pregnancy and 6 visits post-partum). Pregnant women recruited during their first trimester (0-13 weeks of gestation) are interviewed once during each trimester at the OBGYN clinics of the selected health care facilities in Qatar and Lebanon. Prenatal assessments consist of anthropometric measurements and administration of questionnaires for the collection of maternal factors including dietary intake and supplement use before pregnancy, as well as dietary intake, supplement use, and lifestyle practices during pregnancy. In addition, sociodemographic and socioeconomic characteristics as well as household food security data are collected once during the first trimester of pregnancy. A blood sample for biochemical analysis of several biomarkers is also withdrawn once from pregnant women in their first trimester. Additionally, questions to assess maternal exposure, knowledge, attitudes, and intentions towards infant feeding practices are asked during the third trimester visit. After delivery, a member of the research team obtains delivery and birth outcome data from hospital records (after subject’s consent). Postnatal visits for the assessment of infants and young children take place when the child is 4, 6, 9, 12, 18, and 24 months old. The post-natal visits are scheduled during routine visits with the child’s physician in the clinic (or possibly at home). Postnatal assessments consist of anthropometric measurements and assessment of young child feeding practices (breastfeeding and complementary feeding practices), dietary intake, supplement use, and the eating environment at home. Moreover, post-partum assessment of mothers also takes place at each postnatal visit, and consists of anthropometric measurements and collection of dietary intake data, supplement use, lifestyle practices, and household food security.

### Data collection

Data collection for this study includes multi-component questionnaires, anthropometric measurements, as well as biochemical assessments. In addition, delivery and birth outcomes are obtained from hospital records. Below is a brief description of the data collection tools and methods used in the study. Further details are found in Additional file 1.

#### Questionnaires used in data collection

Multicomponent questionnaires were developed to be used for the collection of data. The content validity of these questionnaires was confirmed by a panel of experts consisting of two neonatal physicians, one nutrition epidemiologist, and two clinical nutritionists. The original version of the questionnaires was written in English, translated to Arabic then back-translated to English to ensure parallel-form reliability. The original and the back translated questionnaire versions were reviewed for consistency in meaning by two bilingual experts. A pilot testing of these questionnaires was conducted to ensure that the wording was appropriate and would yield the required data. These questionnaires are administered by trained research nutritionists through face-to-face interviews.

##### Maternal sociodemographic and lifestyle questionnaire

This questionnaire is used to collect information about the mother’s age, place of residence, occupation, education, living arrangements, income, consanguinity, and lifestyle practices during and after pregnancy (physical activity, cigarette and narghili smoking, and alcohol intake). In addition, a section on maternal knowledge and attitudes towards infant feeding practices and intentions to breastfeed is included in this questionnaire.

##### Dietary intake assessment questionnaire of the mothers

Dietary intake of mothers is evaluated using a 98-item culture-specific food frequency questionnaire (FFQ) as well as multiple-pass 24-h recalls. Supplement use is also assessed. Details about the development of the FFQ are described elsewhere [54]. The FFQ was originally developed in Lebanon; hence in order for it to be also used in Qatar, a panel of experts, including a nutritionist, revised the FFQ in order to adapt it to the Qatari dietary habits. Moreover, due to religious and cultural specificities of Qatar, whereby Muslims are not expected to consume any alcohol, asking questions regarding alcohol consumption to Muslim women may possibly create a negative reaction; hence the section on “Alcoholic Beverages” in the FFQ is asked only to non-Muslims. The FFQ to be completed by study participants during the first visit (1^st^ trimester) refers to food intake during the three months-period prior to the participant’s pregnancy while the remaining FFQs refer to the three months preceding the interview.

In addition to the FFQs, eight 24-h dietary recalls are administered (three during pregnancy and five post-partum). In this study, we opted to use the United States Department of Agriculture’s Multiple Pass Food Recall (MPR). The MPR has been shown to attenuate the limitation of recall bias arising from a 24-h dietary recall [55, 56].

##### Sociodemographic/economic characteristics of the household

In addition to maternal factors, data about the father’s as well as the household’s characteristics are also collected and include paternal education, occupation, income, crowding index, and the household food security. The latter is examined using the validated Arabic version of the household food insecurity access scale (HFIAS) [57].

##### Infant and young child feeding practices and dietary intake

Child breastfeeding and complementary feeding practices is assessed according to WHO indicators [58] and include questions related to breastfeeding and complementary feeding. In addition, child dietary intake and supplement use are evaluated using a 24-h recall with the mother as proxy (once at each of the six visits after birth).

#### Anthropometric assessment

In addition to data collected by questionnaires, anthropometric measurements for mothers, infants, and young children are obtained.

##### Anthropometric assessment for mothers

Maternal anthropometric assessments follow standard techniques and encompass height, weight, waist circumference in addition to percentage body fat. The latter is assessed by bioelectrical impedance using an electrical bio-impedance analyzer (Imp DF50, ImpediMed Limited, Brisbane, QLD, Australia).

##### Anthropometric assessment of infants and young children

For infants and young children, anthropometric measurements are taken at each of the six visits after birth and include head circumference, length, weight, and mid-upper arm circumference (MUAC).

#### Biochemical and blood pressure assessments

Fasting maternal blood samples are obtained once in the first trimester to assess maternal micronutrient status. Blood samples are collected by a certified phlebotomist and in appropriate test tubes (with or without ethylenediaminetetraacetic acid [EDTA]) depending on the biomarker to be analyzed. Test tubes are temporarily stored in iceboxes until centrifugation and analysis. The biomarkers that are examined and their corresponding analytical methods to be used are:

- Hemoglobin (Photometry)
- Ferritin (Electrochemiluminescent assay, Roche Cobas 6000)
- Folate (Electrochemiluminescent assay, Roche Cobas 6000)
- Vitamin B12 (Electrochemiluminescent assay, Roche Cobas 6000)
- Vitamin A (High-Performance Liquid Chromatography)
- Vitamin D (Electrochemiluminescent assay, Roche Cobas 6000)
- Lead (Atomic Absorption Spectrometry)
- Zinc (Inductively Coupled Plasma Mass Spectrometry (ICPMS))

Blood pressure is measured once at each of the three visits during pregnancy, using a mercury sphygmomanometer with the subjects seated and after a 5-min rest.

#### Delivery and birth outcome data

Delivery and birth outcome data are obtained from hospital records at the hospital in which the pregnant woman gives birth (if subject provides consent). This data includes: occurrence of complications during pregnancy, delivery method, occurrence of complications during delivery, gestational age, date of delivery, sex of the newborn and his/her birth weight, length, and head circumference measures.

### Reliability of data collection

Given the fact that data collection is carried out by a team of field workers in different sites and in two different countries (Qatar and Lebanon), various quality assurance activities were implemented to ensure the reliability of data collection: Instruments used in data collection such as weighing scales, stadiometers, measuring tapes, etc. were carefully chosen to be identical in all sites and to present high quality reliability data. A five-day data collection training program was developed by the principal investigators in both countries and certification of participation in this program was given to all research assistants involved in data collection. A detailed Operations Manual describing the various steps to be followed before, during, and after data collection was prepared and disseminated to the research team. In this manual, checklists for before and after each visit as well a section on Frequently Asked Questions (FAQs) were included. In both countries, weekly meetings between the principal investigators and personnel involved in data collection were held, during which probing and interviewing techniques were standardized among research assistants in order to minimize interviewer bias [59]. Routine visits of the principal investigators are executed to each site to observe data collection activities. In addition, periodic travels between Doha and Beirut are taking place to discuss emerging situation and standardize procedures.

### Statistical analysis and determination of sample size

The data collected are entered and analyzed using the Statistical Package for the Social Sciences (SPSS) version 22.0. Normality of the variables is examined using standard tests and, when needed, appropriate transformation is applied. For variables with normal distribution, the mean, standard deviation, and Student’s *t* test is used. For variables with distribution differing from normal, the median, interquartile range and Mann-Whitney test is used. The frequency and the chi-square test (*χ*^2^) or Fisher’s exact test is used for categorical variables.

Six main categories of variables are generated: maternal factors, birth outcomes, breastfeeding, complementary feeding, growth patterns of the child, and household food insecurity. The associations among these variables are examined using linear regression when the outcome considered is a continuous variable (such as weight at birth) and logistic regression when the outcome studied is categorical (such as term versus preterm deliveries). The two main dependent variables in this study are related to birth outcomes and growth patterns of the young child. However, while a certain variable is treated as an outcome measure in a regression model, this same variable is used an independent variable in another regression model to help predict other variables. For example, while weight at birth is treated as an outcome variable and is regressed on dietary and lifestyle characteristics of the pregnant woman, this same variable (weight at birth) is used as an independent variable in another regression model to predict obesity and overweight at the age of 2 years. All potential confounders are cross-tabulated against the outcome variables, and only those showing an association (*p* < 0.20) are taken to the multivariable analyses. Modeling of the regression analysis includes a combination of forward and backward regressions. The level of statistical significance is set at *P* < 0.05 for all tests.

One type of analysis that is frequently carried out to assess the strength and direction of a correlation between two numerical variables is correlation using Pearson’s correlation coefficient *r* such as the association between macronutrients consumption during pregnancy and child’s BMI z-score. Pearson’s *r* in its absolute value ranges from 0 to 1 and the following categorization has been suggested by Cohen (1977) [60]: 0-0.2 (weak association), 0.21-0.40 (weak-moderate), 0.41-0.60 (moderate to strong), 0.61-0.80 (strong), and 0.80-1.00 (very strong association). Using the Power Analysis and Sample Size (PASS) software version 11 with a sample size of around 200, a correlation as small as 0.20 would be detected. Correlations smaller than 0.2 would be too small to be considered clinically significant. Another analysis used in this study is linear regression whereby the outcome variable is regressed on a number of independent variables to help explain and predict the outcome. Power analysis and sample size determination for regression analysis are complicated and often not easy to explain since the effect size is determined by the global slope of the model rather than the different individual slopes. A simpler alternative is to consider 10 observations for each independent variable. Hence, with the 200 identified in the earlier analysis it can be argued that a linear regression can be created with as many as 20 independent variables. It is not expected that the regression model in this study would include as many independent variables. It, therefore, appears that the sample size of 200 is adequate for this study. Previous cohort studies following pregnant women and their children throughout pregnancy and young childhood have shown an attrition rate of 15 % [61]. Hence, the target sample size to allow for lost to follow-up throughout the study period is a total of 250 pregnant women in each of Qatar and Lebanon. This lost to follow up may incur undue biases of the results. To address these biases, it is important to examine whether subjects who dropped out differed significantly from those who remained in the cohort with regard to the main exposures considered in the study. In the case where both of these groups are similar, then it could be assumed that participants, on whom the main analysis and conclusions were based, are to a large extent representative of all participants enrolled into the study. However in the case where the two groups are different in relation to important exposures, then other alternative strategies are explored such as imputing outcomes to participants lost to follow up [62]. Given the aforementioned challenges with the lost to follow up biases, every effort is exerted to attempt to achieve the maximum retention rate possible in this cohort.

### Development of evidence-based country-specific nutrition and lifestyle guidelines for pregnant women and young children in Lebanon and Qatar

**Fig. 2:**
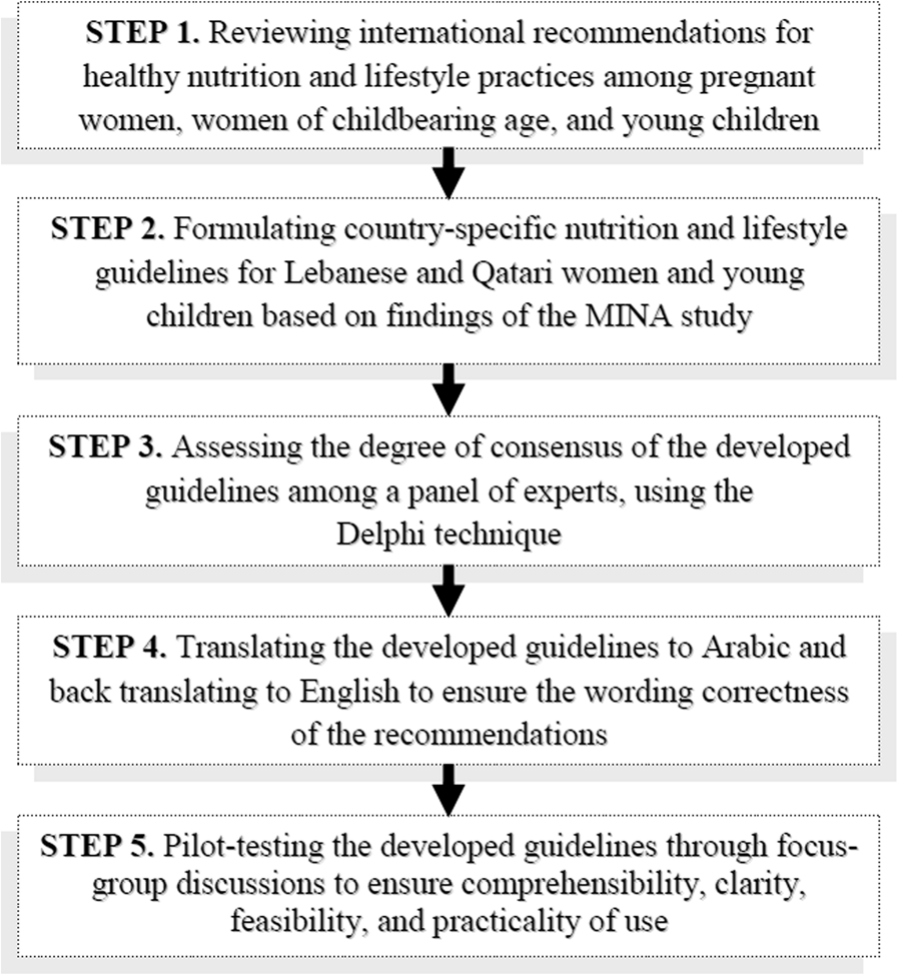
The steps for the development of the evidence based country specific nutrition and lifestyle guidelines using the Delphi technique

The development of the evidence-based country-specific nutrition and lifestyle guidelines will follow a multi-step process including examination of existing international evidence, contextualizing the evidence using results from the MINA cohort study, formulation of the guidelines, and assessment of the degree of consensus among a panel of experts regarding the proposed guidelines using the Delphi technique (Fig. 2).

A thorough and structured literature search of international evidence for healthy nutrition and lifestyle practices of pregnant women and young children will be conducted using defined keywords and inclusion criteria. The quality and strength of this evidence will then be graded according to published standards. A merge between the results of the literature search and the findings of this study will be performed to develop guidelines that are, on one hand, in line with international evidence and are, on the other hand, rooted within the Qatari and Lebanese contexts. A panel of experts will be formed to examine, not only the rigor of the scientific evidence of the developed guidelines, but also their consistency, clinical relevance, and cultural adaptability to each of the two countries. This working group will include, in addition to the researchers involved in this study, members of the public (a few participants in this study could be invited to reflect the general public opinion), delegates from the Ministry of Health, officials from the regional WHO office, and representatives from the food industry. The Delphi approach, which is a qualitative, systematic, and interactive research method, will be used to measure the consensus of the formed panel on the developed guidelines. This approach is described in details in Jones et al. (1995) [63]. The developed guidelines, formulated in the English language, will then be translated to Arabic and back-translated to make sure that the specific recommendations are correctly conveyed to the reader. Before dissemination, these guidelines will be also pilot-tested through focus-group discussions to ensure their comprehensibility, clarity, feasibility, and practicality of use in Lebanon and Qatar.

## Discussion

We describe the design for a prospective three year cohort study of pregnant women and their children investigating nutrition and lifestyle exposures and their relationship with growth patterns in two countries of varying socioeconomic levels in the MENA region: Lebanon and Qatar. This mother and child cohort study is a pioneer endeavor in the Middle Eastern region to investigate the association of nutrition imbalances early in life with birth outcomes, growth patterns, as well as early determinants of NCDs. Not only does this cohort study pave the way for national culture-specific interventions for pregnant women and young children in Lebanon and Qatar, but it also leads to the birth of the first prospective cohort databases in these two countries. Existing studies in the region have focused primarily on nutritional and lifestyle determinants of NCDs in adult populations. Moreover, most of these studies are limited by their cross-sectional design nature that may not imply causality and does not allow for establishing temporal associations between exposure and disease.

Embarking on a prospective cohort study, such as the MINA, is timely given the accumulating evidence for the role of early nutrition on growth and development as well as on the risk of NCDs later in life. Combatting malnutrition, especially in early life, has gained international momentum and has reached the top of global health as well as political agendas. Hosting significant proportions of stunting and wasting coupled to surging rates of NCDs, national authorities in both Qatar and Lebanon have called to address maternal and child nutrition. Qatar’s National Development Strategy (2011-2016) [64] has explicitly emphasized the integration of early prevention and intervention of obesity and other NCDs into different aspects of the health care system, with special focus on improving maternal and child health. More specifically, and in line with the Qatar’s National Development Strategy, are two main objectives of the Qatar National Nutrition and Physical Activity Action Plan (2011-2016) [65]: 1) promoting optimal maternal health care and 2) ensuring proper infant and young child development. In Lebanon, in its latest strategic plan, the Ministry of Public Health listed “development of targeted programs to improve maternal and child health” as a national priority. In addition, “prevention of non-communicable diseases” was also among the developmental objectives included in this strategic plan [66]. The MINA project, therefore, addresses global as well as national calls to improve health and nutrition during the first 1000 days of life. Results of this study will fill a critical knowledge gap in these two countries, providing baseline information about maternal and infant nutritional status and dietary practices. The developed guidelines will promote balanced nutrition during pregnancy; hence, providing essential nutrients to the growing fetus. In addition, the guidelines will target feeding practices, particularly breastfeeding and complementary feeding, during the first two years of life, to ensure proper young child growth and development. Providing adequate nutrition during these 1000 days will ultimately contribute to curbing the growing epidemic of NCDs in Qatar and Lebanon.

The MINA study protocol is an example of how collaborations between countries of different socio-economic levels within the same region may improve research efficiency in the field of early nutrition thus potentially leading to healthier pregnancies, mothers, infants, and children. Financial abilities of Qatar together with the scientific rigor and expertise in both Qatar and Lebanon are joined together to foster research infrastructures and give rise to potential solutions for nutrition and public health issues burdening the health systems of both countries. Being a member of the GCC, specific findings pertaining to Qatar may be applicable to neighboring countries of the Arab Gulf. On the other hand, study findings arising from Lebanon will be relevant and applicable to neighboring Middle Eastern Arab countries with similar economic and cultural background.

Distinctive features of the MINA cohort protocol include the multi-sectoral collaborations and multi-disciplinary research team as well as the comprehensive assessment of nutrition and lifestyle exposures. Collaborations between public, private and academic institutions in both countries were instrumental to put together the logistics for launching the MINA cohort. The Supreme Council of Health in Qatar, Qatar University, the American University of Beirut in Lebanon and Primary Health Care Centers in both Countries were linked in a network to facilitate subjects’ recruitment, data collection, and follow up; always ensuring the utmost respect and compliance with the regulations of the ethical review bodies overlooking the conduct of the study. Furthermore, the team of researchers involved in the MINA study combines multidisciplinary skills with expertise in obstetrics, neonatal medicine, nutrition, and dietetics, as well as nutrition epidemiology. An additional feature of the MINA is the regular and systematic monitoring of dietary intake, lifestyle practices, body composition, and growth of mothers and their offspring starting from the first trimester of gestation and continuing up to two years after birth. With this follow-up, this study offers a rich collection of predictor variables, confounding factors, and proxy outcomes for later prediction of disease risk.

## Additional file

Additional file 1: Details on Data Collection. (DOCX 50 kb)

## Abbreviations

AUBMC: American University of Beirut Medical Center
BMI: body mass index
CI: confidence interval
DALYs: disability adjusted life-years
EDTA: ethylenediaminetetraacetic acid
FFQ: food frequency questionnaire
GCC: gulf cooperation council
HFIAS: household food insecurity access scale
IPAQ: international physical activity questionnaire
IRB: Institutional Review Board
MENA: Middle East and North Africa
MPR: multiple pass food recall
MUAC: mid-upper arm circumference
NCDs: non-communicable diseases
OBGYN: obstetrics and gynecology
OR: odds ratio
PASS: power analysis and sample size
PHCC: primary health care corporation
PPAQ: pregnancy physical activity questionnaire
SPSS: statistical package for the social sciences
UNICEF: United Nations Children’s Fund
US: United States
WHO: World Health Organization

## References

[1] Black RE, Victora CG, Walker SP, Bhutta ZA, Christian P, de Onis M (2013). Maternal and child undernutrition and overweight in low-income and middle-income countries. Lancet.

[2] Siega-Riz AM, Viswanathan M, Moos MK, Deierlein A, Mumford S, Knaack J (2009). A systematic review of outcomes of maternal weight gain according to the Institute of Medicine recommendations: birthweight, fetal growth, and postpartum weight retention. Am J Obstet Gynecol.

[3] Li N, Liu E, Guo J, Pan L, Li B, Wang P (2013). Maternal prepregnancy body mass index and gestational weight gain on pregnancy outcomes.

[4] Lau EY, Liu J, Archer E. Maternal weight gain in pregnancy and risk of obesity among offspring: a systematic review. 2014;2014:524939. doi:10.1155/2014/524939. 10.1155/2014/524939 PMC4202338 25371815

[5] Gaillard R, Steegers EA, Franco OH, Hofman A, Jaddoe VW. Maternal weight gain in different periods of pregnancy and childhood cardio-metabolic outcomes. The Generation R Study. Int J Obes (Lond). 2015;39(4):677-85. doi:10.1038/ijo.2014.175. 10.1038/ijo.2014.175 25287752

[6] Augustyniak RA, Singh K, Zeldes D, Singh M, Rossi NF (2010). Maternal protein restriction leads to hyperresponsiveness to stress and salt-sensitive hypertension in male offspring. Am J Physiol Regul Integr Comp Physiol.

[7] Ramadan WS, Alshiraihi I, Al-karim S (2013). Effect of maternal low protein diet during pregnancy on the fetal liver of rats. Ann Anat.

[8] Khan IY, Dekou V, Douglas G, Jensen R, Hanson MA, Poston L (2005). A high-fat diet during rat pregnancy or suckling induces cardiovascular dysfunction in adult offspring. Am J Physiol Regul Integr Comp Physiol.

[9] Bispham J, Gardner DS, Gnanalingham MG, Stephenson T, Symonds ME, Budge H (2005). Maternal nutritional programming of fetal adipose tissue development: differential effects on messenger ribonucleic acid abundance for uncoupling proteins and peroxisome proliferator-activated and prolactin receptors. Endocrinology.

[10] Herrera E, Ortega-Senovilla H (2014). Lipid metabolism during pregnancy and its implications for fetal growth. Curr Pharm Biotechnol.

[11] Maslova E, Rytter D, Bech BH, Henriksen TB, Olsen SF, Halldorsson TI. Maternal intake of fat in pregnancy and offspring metabolic health - A prospective study with 20 years of follow-up. Clin Nutr. 2015. doi:10.1016/j.clnu.2015.03.018. 10.1016/j.clnu.2015.03.018 25933442

[12] Ronnenberg AG, Goldman MB, Chen D, Aitken IW, Willett WC, Selhub J (2002). Preconception homocysteine and B vitamin status and birth outcomes in Chinese women. Am J Clin Nutr.

[13] de la Calle M, Usandizaga R, Sancha M, Magdaleno F, Herranz A, Cabrillo E (2003). Homocysteine, folic acid and B-group vitamins in obstetrics and gynaecology. Eur J Obstet Gynecol Reprod Biol.

[14] Scholl TO (2005). Iron status during pregnancy: setting the stage for mother and infant. Am J Clin Nutr.

[15] Furness D, Fenech M, Dekker G, Khong TY, Roberts C, Hague W (2013). Folate, vitamin B12, vitamin B6 and homocysteine: impact on pregnancy outcome. Matern Child Nutr.

[16] Gadgil M, Joshi K, Pandit A, Otiv S, Joshi R, Brenna JT (2014). Imbalance of folic acid and vitamin B12 is associated with birth outcome: an Indian pregnant women study. Eur J Clin Nutr.

[17] Bora R, Sable C, Wolfson J, Boro K, Rao R (2014). Prevalence of anemia in pregnant women and its effect on neonatal outcomes in Northeast India. J Matern Fetal Neonatal Med.

[18] Victora CG, de Onis M, Hallal PC, Blossner M, Shrimpton R (2010). Worldwide timing of growth faltering: revisiting implications for interventions. Pediatrics.

[19] Bhutta ZA, Ahmed T, Black RE, Cousens S, Dewey K, Giugliani E (2008). What works? Interventions for maternal and child undernutrition and survival. Lancet (London, England).

[20] Bhutta ZA, Das JK, Rizvi A, Gaffey MF, Walker N, Horton S (2013). Evidence-based interventions for improvement of maternal and child nutrition: what can be done and at what cost?. Lancet (London, England).

[21] Horta BL, Victora CG (2013). Long-term effects of breastfeeding-a systematic review.

[22] Ramakrishnan U, Imhoff-Kunsch B, Martorell R (2014). Maternal nutrition interventions to improve maternal, newborn, and child health outcomes.

[23] Forsdahl A (1977). Are poor living conditions in childhood and adolescence an important risk factor for arteriosclerotic heart disease?. Br J Prev Soc Med.

[24] Barker DJ, Winter PD, Osmond C, Margetts B, Simmonds SJ (1989). Weight in infancy and death from ischaemic heart disease. Lancet (London, England).

[25] Barker DJ (1998). In utero programming of chronic disease. Clin Sci (Lond).

[26] Horton R (2008). Maternal and child undernutrition: an urgent opportunity. Lancet (London, England).

[27] Save the Children. Nutrition in the first 1000 days: State of the world’s mothers 2012 Save the Children Fund. 2012. http://www.savethechildren.org/atf/cf/%7B9def2ebe-10ae-432c-9bd0-df91d2eba74a%7D/STATEOFTHEWORLDSMOTHERSREPORT2012.PDF. Accessed 26 Jun 2015.

[28] Arenz S, Ruckerl R, Koletzko B, von Kries R (2004). Breast-feeding and childhood obesity--a systematic review. Int J Obes Relat Metab Disord.

[29] Horta BL (2007). Evidence on the long-term effects of breastfeeding.

[30] Oddy W, Li J, Robinson M, Whitehouse A (2012). The Long-Term Effects of Breastfeeding on Development.

[31] Shields L, Mamun AA, O'Callaghan M, Williams GM, Najman JM (2010). Breastfeeding and obesity at 21 years: a cohort study. J Clin Nurs.

[32] Ip S, Chung M, Raman G, Chew P, Magula N, DeVine D (2007). Breastfeeding and maternal and infant health outcomes in developed countries. Evid Rep Technol Assess.

[33] Atladottir H, Thorsdottir I (2000). Energy intake and growth of infants in Iceland-a population with high frequency of breast-feeding and high birth weight. Eur J Clin Nutr.

[34] Spyrides MH, Struchiner CJ, Barbosa MT, Kac G (2008). Effect of predominant breastfeeding duration on infant growth: a prospective study using nonlinear mixed effect models. J Pediatr (Rio J).

[35] Singhal A, Lanigan J (2007). Breastfeeding, early growth and later obesity. Obes Rev.

[36] Kelishadi R, Farajian S (2014). The protective effects of breastfeeding on chronic non-communicable diseases in adulthood: A review of evidence. Adv Biomed Res.

[37] Huh SY, Rifas-Shiman SL, Taveras EM, Oken E, Gillman MW (2011). Timing of solid food introduction and risk of obesity in preschool-aged children. Pediatrics.

[38] Seach KA, Dharmage SC, Lowe AJ, Dixon JB (2010). Delayed introduction of solid feeding reduces child overweight and obesity at 10 years. Int J Obes (Lond).

[39] WHO. Regional Strategy on Nutrition 2010-2019 and Action Plan. World Health Organization Regional Office for the Eastern Mediterranean (WHO-EMRO). World Health Organization. World Health Organization, Cairo, Egypt. 2011. http://www.emro.who.int/nutrition/nutrition-infocus/in-focus.html. Accessed 6 May 2015.

[40] Len-Cava N, Lutter C, Ross J, Martin L. Quantifying the benefits of breastfeeding: a summary of the evidence. Pan American Health Organization, Washington DC. 2002. http://www.ennonline.net/quantifyingbenefitsbreastfeeding2. Accessed 4 May 2015.

[41] de Onis M, Blossner M, Borghi E (2010). Global prevalence and trends of overweight and obesity among preschool children. Am J Clin Nutr.

[42] Ng M, Fleming T, Robinson M, Thomson B, Graetz N, Margono C (2014). Global, regional, and national prevalence of overweight and obesity in children and adults during 1980-2013: a systematic analysis for the Global Burden of Disease Study 2013. Lancet (London, England).

[43] Atinmo T, Mirmiran P, Oyewole OE, Belahsen R, Serra-Majem L (2009). Breaking the poverty/malnutrition cycle in Africa and the Middle East. Nutr Rev.

[44] Musaiger AO, Al-Hazzaa HM (2012). Prevalence and risk factors associated with nutrition-related noncommunicable diseases in the Eastern Mediterranean region. Int J Gen Med.

[45] El-Kogali S, Krafft C. Expanding Opportunities for the Next Generation: Early Childhood Development in the Middle East and North Africa. World Bank Publications; 2015.

[46] Ng SW, Zaghloul S, Ali HI, Harrison G, Popkin BM (2011). The prevalence and trends of overweight, obesity and nutrition-related non-communicable diseases in the Arabian Gulf States. Obes Rev.

[47] Howard JJ (2014). Medical devices and the Middle East: market, regulation, and reimbursement in Gulf Cooperation Council states. Med Devices (Auckl).

[48] Sibai A, Hwalla N, Adra N, Rahal B (2003). Prevalence and covariates of obesity in Lebanon: findings from the first epidemiological study. Obes Res.

[49] UNICEF, World Bank, WHO. Joint malnutrition dataset on child malnutrition estimates. 2014. http://www.who.int/nutgrowthdb/estimates/en/. Accessed 22 Apr 2015.

[50] WHO. Noncommunicable diseases. Country profiles. World Health Organization. World Health Organization. 2014a. http://www.who.int/nmh/publications/ncd-profiles-2014/en/. Accessed 2 May 2015.

[51] WHO. Global Health Observatory Data Repository: Obesity data by country. World Health Organization. World Health Organization. 2014b. http://www.who.int/gho/ncd/risk_factors/overweight/en/. Accessed 4 June 2015.

[52] Seidell JC (2000). Obesity, insulin resistance and diabetes--a worldwide epidemic. Br J Nutr.

[53] Nasreddine L, Naja F, Chamieh MC, Adra N, Sibai AM, Hwalla N (2012). Trends in overweight and obesity in Lebanon: evidence from two national cross-sectional surveys (1997 and 2009). BMC Public Health.

[54] Nasreddine L, Hwalla N, Sibai A, Hamze M, Parent-Massin D (2006). Food consumption patterns in an adult urban population in Beirut, Lebanon. Public Health Nutr.

[55] Moshfegh AJ, Rhodes DG, Baer DJ, Murayi T, Clemens JC, Rumpler WV (2008). The US Department of Agriculture Automated Multiple-Pass Method reduces bias in the collection of energy intakes. Am J Clin Nutr.

[56] Raper N, Perloff B, Ingwersen L, Steinfeldt L, Anand J (2004). An overview of USDA’s dietary intake data system. J Food Composition Anal.

[57] Naja F, Hwalla N, Fossian T, Zebian D, Nasreddine L. Validity and reliability of the Arabic version of the Household Food Insecurity Access Scale in rural Lebanon. Public Health Nutr. 2014:1-8. doi:10.1017/s1368980014000317. 10.1017/S1368980014000317 PMC10271416 24702865

[58] WHO. WHO: Indicators for assessing infant and young child feeding practices. Part 1 Definitions. World Health Organization. Geneva: WHO. 2008a. http://www.who.int/maternal_child_adolescent/documents/9789241596664/en/.

[59] Whitney CW, Lind BK, Wahl PW (1998). Quality assurance and quality control in longitudinal studies. Epidemiol Rev.

[60] Cohen J. Statistical power analysis for the behavioral sciences (rev. Lawrence Erlbaum Associates, Inc; 1977.

[61] Gracie SK, Lyon AW, Kehler HL, Pennell CE, Dolan SM, McNeil DA (2010). All Our Babies Cohort Study: recruitment of a cohort to predict women at risk of preterm birth through the examination of gene expression profiles and the environment. BMC Pregnancy Childbirth.

[62] Kristman V, Manno M, Cote P (2004). Loss to follow-up in cohort studies: how much is too much?. Eur J Epidemiol.

[63] Jones J, Hunter D (1995). Consensus methods for medical and health services research. BMJ.

[64] Qatar General Secretariat for Development Planning (2011). Qatar’s National Development Strategy 2011-2016.

[65] NHS. National Health Strategy. Qatar National Nutrition and Physical Activity Plan (2011-2016). National Health Strategy. 2011. https://extranet.who.int/nutrition/gina/sites/default/files/QAT%202011%20National%20Nutrition%20and%20Physical%20Activity%20Action%20Plan.pdf. Accessed 22 May 2015.

[66] MOPH. The MOH Strategic Plan. The Ministry of Public Health, Lebanon. 2007. http://www.moph.gov.lb/media/documents/themohstrategyplanmodified.pdf. 2012. Accessed 26 Jun 2015.

