# Supplementary material for: Study protocol: Mother and Infant Nutritional Assessment (MINA) cohort study in Qatar and Lebanon
Source: BMC Pregnancy Childbirth. 2016 May 4;16:98. doi: 10.1186/s12884-016-0864-5 (PMC4855720; doi:10.1186/s12884-016-0864-5)
Supplement: Additional file 1: — Details on Data Collection. (DOCX 50 kb) [file 12884_2016_864_MOESM1_ESM.docx]

**Appendix A**

**Details on Data Collection**

Data collection for this study includes multi-component questionnaires, anthropometric measurements, as well as biochemical assessments. In addition delivery and birth outcomes will be obtained from hospital records.

Questionnaire data collection

Multicomponent questionnaires were developed to be used for the collection of data. The content validity of these questionnaires was confirmed by a panel of experts consisting of two neonatal physicians, one nutrition epidemiologist, and two clinical nutritionists. The original version of the questionnaires was written in English; yet, since the questionnaires are planned to be administered to study participants in their native Arabic language, questionnaires were translated to Arabic then back-translated to English to ensure parallel-form reliability. The original and the back translated questionnaire versions were reviewed for consistency in meaning by two bilingual experts. A pilot testing of these questionnaires was conducted to ensure that the wording was appropriate and would yield the required data. These questionnaires will be administered by trained research nutritionists through face-to-face interviews. Regular meetings will be held among all research staff involved in field work in order to standardize the methods of data collection and decrease inter-rater variability.

*Maternal sociodemographic and lifestyle questionnaire*

This questionnaire will be used to collect information about:

- Maternal age, place of residence, occupation, education, living arrangements, income, consanguinity.
- Maternal lifestyle practices (physical activity, cigarette and narghili smoking, and alcohol intake) during and after pregnancy
  - Maternal physical activity levels: will be assessed using questions and scoring methods set by the Pregnancy Physical Activity Questionnaire (PPAQ) [1] for assessment of physical activity during pregnancy, and by the short International Physical Activity Questionnaire (IPAQ) for assessment of post-partum physical activity levels [2]. These questionnaires were revised to adapt to the local context in Lebanon and Qatar. For instance, examples related to mowing the lawn were deleted as these activities are not common to women in Lebanon or Qatar. Furthermore, in two questions asking about walking while carrying things, the concept of carrying “*something heavier than a 1 gallon milk jug’* was modified to state “*something heavier than a 4 kg bag of rice”,* given that milk in Lebanon and Qatar is not sold as one gallon milk jugs.
  - Maternal cigarette smoking dose: will be assessed based on criteria defined by Bachir and Chaaya as: mild smoking: 1–9 cigarettes/day; moderate smoking: 10–20 cigarettes/day; and heavy smoking: > 20 cigarettes/day [3].
  - Maternal narghile smoking dose: will be assessed based on criteria defined by Tamim et al., [4] as follows: mild smoking: ≤ 1 narghile/day (this refers to instances when the episode of smoking lasts for periods less than the average duration of smoking a narghile (10 - 30 minutes) or when a few puffs of the narghile are taken) and moderate smoking: > 1 narghile/day.
- Maternal exposure, knowledge, and attitudes towards infant feeding practices as well as intentions to breastfeed (based on assessment tools proposed by Tarrant and Dodgson, 2007 [5]; Kavanagh et al., 2012 [6]; Grossman et al., 1990 [7]; De La Mora et al., 1999 [8]; Nommsen-Rivers and Dewey, 2009 [9]).

*Dietary intake assessment questionnaire*

Dietary intake of mothers will be evaluated using a culture-specific food frequency questionnaire as well as multiple-pass 24-hour recalls. Supplement use will also be assessed.

- Food Frequency Questionnaire (FFQ): Dietary intake of participant women will be assessed three times during pregnancy and twice after delivery, using an 89-item semi-quantitative FFQ adapted from the dietary instrument used in the total diet study of adult urban population in Beirut, Lebanon [10]. Further details about the development of the original questionnaire are described elsewhere [10]. The first FFQ refers to food intake during the three-months period prior to the participant’s pregnancy while the remaining FFQs refer to the three months preceding the interview. Food items listed in the FFQ were grouped into 24 food categories, namely ‘cereals and other cereal-based products’, ‘pasta and other cereals’, ‘potatoes and potato-based products’, ‘vegetables’, ‘fruits’, ‘fruit juices’, ‘meat and cured meat’, ‘offals’, ‘poultry’, ‘eggs’, ‘fish and seafood’, ‘pulses, nuts and seeds’, ‘milk and dairy products’, ‘yogurt and yogurt-based products’, ‘pizzas and pies’, ‘mixed dishes’, ‘fats and oils (added on breads)’, ‘fats and oils (used in frying)’, ‘sugar and sugar derivatives’, ‘cakes and pastries’, ‘honey, jam, molasses and halawah (a sesame seed-based sweet)’, ‘alcoholic beverages’, ‘non-alcoholic beverages’, and a ‘miscellaneous’ food category. Food items under each category were chosen to provide comprehensive representation of the Lebanese diet. Where possible, foods were separated into low fat and whole fat groups. The reference portions of the two-dimensional food portion visual (Millen and Morgan, Nutrition Consulting Enterprises, Framingham, Massachusetts, United States) and common household food portions will be used to assist participants in estimating the proper quantities of their dietary intake. Participants will be asked to recall how often, on average, they consume the specified portion size of every food item on a ‘per day’, ‘per week’, ‘per month’, or ‘never’ basis. Portion sizes, consumed from each food item, will be then converted to daily portion intake. Daily energy and nutrient intakes will be computed by multiplying the daily intake by the nutrient content of the specified portion size; using the food composition tables provided by the Nutritionist Pro software (version 5.1.0, 2014, First Fata Bank, Nutritionist Pro, San Bruno, CA) as well as food composition tables of Middle Eastern foods for local and traditional dishes [10].

For use in Qatar, the developed FFQ was revised by a panel of experts, including a nutritionist, in order to adapt it to dietary habits in Qatar. This FFQ was amended to include a few local foods. Moreover, due to religious and cultural specificities of Qatar, whereby Muslims are not expected to consume alcohol and asking questions regarding alcohol consumption to Muslim women may possibly create a negative reaction, the section on “Alcoholic Beverages” will be asked only to non-Muslims.

- 24-hour dietary recall: In addition to the FFQ, eight repeated 24-hour dietary recalls will be administered (three during pregnancy and five post-partum). Compared to the FFQ, the 24-hour dietary recall does not have a fixed list of food items and relies on short term as opposed to the long term memory. In this study we opted to use the United States Department of Agriculture’s Multiple Pass Food Recall (MPR). The MPR has been shown to attenuate the limitation of recall bias arising from a 24-hour dietary recall [11, 12]. During the interview, the interviewer will probe the participant more than once in an effort to retrieve forgotten eating occasions and foods. Estimation of energy and nutrient intake will be done similar to that of the FFQ as described in the previous section.

*Sociodemographic/economic characteristics of the household*

In addition to maternal factors, data about the father as well as the household will also be collected and will include:

- Paternal education, occupation, and income
- Crowding index (ratio of number of individuals in the household over the number of rooms in the house) which will be used as a proxy for the socioeconomic status of the household, as this index has been shown to be inversely associated with socioeconomic status [13].
- Household food security. The Arabic version of the household food insecurity access scale (HFIAS) will be used to assess food insecurity in the study population (once during pregnancy and once after delivery). This version has been validated for use among Lebanese adults [14]. The recall period for the HFIAS is 4 weeks (30 days) and the HFIAS score will be computed as per Coates et al. [15], where the higher the score obtained, the greater the food insecurity.

*Infant and young child feeding practices and dietary intake*

Child breastfeeding and complementary feeding practices will be assessed according to WHO indicators [16].

- Breastfeeding questions relate to early initiation of breastfeeding, exclusive breastfeeding for 6 months, continued breastfeeding at 1 year, duration of breastfeeding, continued breastfeeding after 2 years, age appropriate breastfeeding, predominant breastfeeding under 6 months, and bottle-feeding.
- Child complementary feeding assessment includes timing of introduction and type of liquids (water and juices), solid, semi-solid and soft foods; minimum dietary diversity; minimum meal frequency; minimum acceptable diet; and consumption of iron-rich or iron-fortified foods.
- Child dietary intake and supplement use will be evaluated using a 24-hour recall with the mother as proxy (once at each of the six visits after birth).

Anthropometric assessment

In addition to the data collected by the questionnaires, anthropometric measurements for mothers, infants, and young children will be obtained.

*Anthropometric assessment for mothers*

Maternal anthropometric assessment will take place in each of the three trimesters in order to assess nutritional status and weight gain. The anthropometric measurements will consist of the following:

- Height will be measured to the nearest 0.1 cm with the subject bare footed, using a stadiometer, once at recruitment in the first trimester (Seca model 213, Germany).
- Weight will be measured to the nearest 0.1 kg with the subject bare footed and in light clothes, using a standard clinical balance (Seca model 770, Germany), at each of the study visits. Pre-pregnancy weight and post-partum weights will be classified and assessed according to the WHO (2000) classification criteria for body mass index (BMI) [17]. Pregnancy weight gain and rate of weight gain will be classified and assessed according to the Institute of Medicine’s BMI criteria for pregnant women [18].
- Waist circumference will be measured to the nearest 0.1 cm with the subject in light clothes, using a flexible non-stretchable measuring tape (Seca model 201, Germany), at each of the study visits except during the second and third trimesters of pregnancy. Waist circumference will be assessed according to the International Diabetes Federation recommended cut-off point for defining abdominal obesity in people of Middle Eastern origin which is ≥ 80 cm for females [19].
- Percent body fat will be measured by bioelectrical impedance using an electrical bio-impedance analyzer (Imp DF50, ImpediMed Limited, Brisbane, QLD, Australia), at each of the visits post-partum. High percent body fat will be defined as > 32% in females [20].

*Anthropometric assessment of infants and young children*

For infants and young children, anthropometric measurements will be taken at each of the six visits after birth and include:

- Head circumference will be measured using a flexible, non-stretchable measuring tape (Seca model 212, Germany). The infant/young child will be placed in a sitting position in the lap of the caregiver. The lower edge of the measuring tape will be placed just above the child's eyebrows, above the ears and around the occipital prominence at the back of the head, to allow the measurement of the maximal head circumference. Head circumference will be used to assess potential developmental or neurological disabilities. Values will be assessed according to WHO [21] criteria as follows: values < 3rd percentile for age or > 97th percentile for age are both indicative of health or developmental risk.
- Length will be measured to the nearest 0.1 cm using a measuring mat (Seca model 210, Germany) placed horizontally on a flat and level surface. The young child's shoes and any head covering will be removed and the child will be placed so he/she is lying down with the head positioned until it touches the head board, the knees held together while being pressed down, and the soles of the feet being flat on the foot piece, toes pointing up at right angles.
- Weight: will be measured to the nearest 0.1 kg using a standard clinical balance (Seca model 770, Germany), set at the tar mother-child function. Diapers will be removed and the child will be stripped to minimal clothing.

Weight and length measurements will be used to assess stunting, wasting, underweight, overweight, and obesity prevalence rates according to the WHO criteria on child growth standards [22].

- Mid-upper arm circumference (MUAC) will be measured to the nearest 0.1 cm using a flexible non-stretchable measuring tape (Seca model 201, Germany).The measurement will be taken at the mid-point between the elbow and the shoulder bones (acromion and olecranon processes) of the left arm with the arm being relaxed and hanging down the side of the body. MUAC will be used to assess nutritional status and will be defined according to the updated WHO and UNICEF [23] criteria as follows: severe under-nutrition (wasting): < 11.5 cm; moderate under-nutrition: 11.5 -12.5 cm; and well nourished: > 12.5 cm.

Biochemical and blood pressure assessments

Fasting maternal blood will be collected once in the first trimester to assess maternal micronutrient status including hemoglobin, ferritin, folate, vitamins B12, A, and D, lead as well as zinc. Collection of blood samples will take place by a certified phlebotomist. Blood samples will be collected in appropriate test tubes (with or without ethylenediaminetetraacetic acid [EDTA]) depending on the biomarker to be analyzed. Test tubes will be temporarily stored in iceboxes until centrifugation and analysis. The following biochemical markers will be assessed according to their respective analytical methods:

- Hemoglobin (Photometry)
- Ferritin (Electrochemiluminescent assay, Roche Cobas 6000)
- Folate (Electrochemiluminescent assay, Roche Cobas 6000)
- Vitamin B12 (Electrochemiluminescent assay, Roche Cobas 6000)
- Vitamin A (High-Performance Liquid Chromatography)
- Vitamin D (Electrochemiluminescent assay, Roche Cobas 6000)
- Lead (Atomic Absorption Spectrometry)
- Zinc (Inductively Coupled Plasma Mass Spectrometry (ICPMS))

Blood pressure will be measured using a mercury sphygmomanometer, with the subjects seated and after a 5-minute rest, once at each of the three visits during pregnancy. Blood pressure measurements will be assessed according to criteria defined by the National Heart, Lung, and Blood Institute of the National Institute of Health [24] as follows: High systolic blood pressure (SBP): > 140 mmHg and high diastolic blood pressure (DBP): > 90 mmHg.

Delivery and birth outcome data

Delivery and birth outcome data will be obtained from hospital records at the hospital in which the pregnant woman gives birth (if subject provides consent). This data includes: maternal pre- and post-delivery weights, oral glucose tolerance test results, occurrence of complications during pregnancy, delivery method, and occurrence of complications during delivery, as well as gestational age, date of delivery, sex of the newborn, and birth weight, length, and head circumference measures.

**References**

1. Chasan-Taber L, Schmidt MD, Roberts DE, Hosmer D, Markenson G, Freedson PS. Development and validation of a Pregnancy Physical Activity Questionnaire. Medicine and science in sports and exercise. 2004;36(10):1750-60.

2. IPAQ. International Physical Activity Questionnaire (IPAQ). 2002. https://sites.google.com/site/theipaq/. Accessed April 12 2012.

3. Bachir R, Chaaya M. Maternal smoking: determinants and associated morbidity in two areas in Lebanon. Maternal and child health journal. 2008;12(3):298-307. doi:10.1007/s10995-007-0242-z.

4. Tamim H, Yunis KA, Chemaitelly H, Alameh M, Nassar AH. Effect of narghile and cigarette smoking on newborn birthweight. BJOG : an international journal of obstetrics and gynaecology. 2008;115(1):91-7. doi:10.1111/j.1471-0528.2007.01568.x.

5. Tarrant M, Dodgson JE. Knowledge, attitudes, exposure, and future intentions of Hong Kong university students toward infant feeding. Journal of obstetric, gynecologic, and neonatal nursing : JOGNN / NAACOG. 2007;36(3):243-54. doi:10.1111/j.1552-6909.2007.00144.x.

6. Kavanagh KF, Lou Z, Nicklas JC, Habibi MF, Murphy LT. Breastfeeding knowledge, attitudes, prior exposure, and intent among undergraduate students. Journal of human lactation : official journal of International Lactation Consultant Association. 2012;28(4):556-64. doi:10.1177/0890334412446798.

7. Grossman LK, Harter C, Hasbrouck C. Testing mothers' knowledge of breastfeeding: instrument development and implementation and correlation with infant feeding decision. Journal of pediatric & perinatal nutrition. 1990;2(2):43-63.

8. Mora Adl, Russell DW, Dungy CI, Losch M, Dusdieker L. The Iowa Infant Feeding Attitude Scale: Analysis of Reliability and Validity1. Journal of Applied Social Psychology. 1999;29(11):2362-80.

9. Nommsen-Rivers LA, Dewey KG. Development and validation of the infant feeding intentions scale. Maternal and child health journal. 2009;13(3):334-42.

10. Nasreddine L, Hwalla N, Sibai A, Hamze M, Parent-Massin D. Food consumption patterns in an adult urban population in Beirut, Lebanon. Public health nutrition. 2006;9(2):194-203.

11. Moshfegh AJ, Rhodes DG, Baer DJ, Murayi T, Clemens JC, Rumpler WV et al. The US Department of Agriculture Automated Multiple-Pass Method reduces bias in the collection of energy intakes. The American journal of clinical nutrition. 2008;88(2):324-32.

12. Raper N, Perloff B, Ingwersen L, Steinfeldt L, Anand J. An overview of USDA's dietary intake data system. Journal of Food Composition and Analysis. 2004;17(3):545-55.

13. Melki IS, Beydoun HA, Khogali M, Tamim H, Yunis KA. Household crowding index: a correlate of socioeconomic status and inter-pregnancy spacing in an urban setting. Journal of epidemiology and community health. 2004;58(6):476-80.

14. Naja F, Hwalla N, Fossian T, Zebian D, Nasreddine L. Validity and reliability of the Arabic version of the Household Food Insecurity Access Scale in rural Lebanon. Public health nutrition. 2014:1-8. doi:10.1017/s1368980014000317.

15. Coates J, Swindale A, Bilinsky P. Household Food Insecurity Access Scale (HFIAS) for measurement of food access: indicator guide. Washington, DC: Food and Nutrition Technical Assistance Project, Academy for Educational Development. 2007.

16. WHO. WHO: Indicators for assessing infant and young child feeding practices. Part 1 Definitions. World Health Organization. Geneva: WHO. 2008a. <http://apps.who.int/iris/bitstream/10665/43895/1/9789241596664_eng.pdf?ua=1&ua=1>.

17. WHO. Obesity: preventing and managing the global epidemic. World Health Organization. vol 894. World Health Organization; 2000.

18. Yaktine AL, Rasmussen KM. Weight Gain During Pregnancy: Reexamining the Guidelines. National Academies Press; 2009.

19. Grundy SM, Cleeman JI, Daniels SR, Donato KA, Eckel RH, Franklin BA et al. Diagnosis and management of the metabolic syndrome: an American Heart Association/National Heart, Lung, and Blood Institute scientific statement: Executive Summary. Critical pathways in cardiology. 2005;4(4):198-203.

20. Lohman TG, Roche AF, Martorell R. Anthropometric standardization reference manual. Champain, IL: Human Kinetics Publishers; 1988.

21. WHO. Global database on child growth and malnutrition. World Health Organization. 2007. <http://www.who.int/nutgrowthdb/en/>. Accessed June 4 2012.

22. WHO. Training course on child growth assessment. World Health Organization. Geneva: WHO. 2008b.

23. WHO, UNICEF. WHO child growth standards and the identification of severe acute malnutrition in infants and children. A Joint Statement by the World Health Organization and the United Nations Children’s Fund2009.

24. NHLBI. High Blood Pressure in Pregnancy. National Heart, Lung, and Blood Institute. National Heart, Lung, and Blood Institute <http://www.nhlbi.nih.gov/health/resources/heart/hbp-pregnancy>. 2012.
